# Risk factors for the development of bronchiolitis obliterans in children after suffering from adenovirus pneumonia

**DOI:** 10.3389/fped.2023.1335543

**Published:** 2024-01-10

**Authors:** Jiahao Yuan, Mengyue Wei, Manke Chen, Ruizhu Wang, Jialing Diao, Man Tian, Deyu Zhao, Meng Chen

**Affiliations:** ^1^ Department of Respiratory Medicine, Children’s Hospital of Nanjing Medical University, Nanjing, China; ^2^ Department of Radiology, Children’s Hospital of Nanjing Medical University, Nanjing, China

**Keywords:** adenovirus pneumonia, bronchiolitis obliterans, children, logistic regression analysis, risk factors

## Abstract

**Introduction:**

Bronchiolitis obliterans (BO) is an irreversible chronic obstructive lung disease in small airways. The aim of this study was to identify the relevant risk factors for the development of BO in children after suffering from adenovirus (ADV) pneumonia.

**Methods:**

An observational cohort study that included 112 children suffering from ADV pneumonia in our institution from March 2019 to March 2020 was performed. We divided the children into a BO group and a non-BO group based on whether they did develop BO or not. Univariate analysis and multivariate logistic regression analysis were applied to identify risk factors for the development of BO. The prediction probability model was evaluated by receiver operating characteristic (ROC) curve analysis.

**Results:**

Twenty-eight children (25%) did develop BO after suffering from ADV pneumonia, while 84 children did not. Respiratory support (OR 6.772, 95% CI 2.060–22.260, *P *= 0.002), extended length of wheezing days (OR 1.112, 95% CI 1.040–1.189, *P *= 0.002) and higher lactic dehydrogenase (LDH) levels (OR 1.002, 95% CI 1.000–1.003, *P *= 0.012) were independently associated with the development of BO. The predictive value of this prediction probability model was validated by the ROC curve, with an area under the curve of 0.870 (95% CI 0.801–0.939, *P *< 0.001), a standard error of 0.035, a maximum Youden's index of 0.608, a sensitivity of 0.929, and a specificity of 0.679.

**Conclusions:**

After suffering an ADV pneumonia, children who have needed respiratory support, had a longer length of wheezing days or had higher LDH levels are more likely to develop BO.

## 1 Introduction

Bronchiolitis obliterans (BO) is an irreversible chronic obstructive lung disease in small airways (1). It is a rare disease with characteristic features of fibrosis of terminal and distal bronchioles. It was first reported and named by German scholar Lange in 1901 and manifests as persistent cough, wheezing, exercise intolerance, and persistent crackles and wheezing on lung auscultation (2). Among these, persistent wheezing is the most common complaint (3). BO can be caused by a variety of factors including severe lower respiratory tract infections, bone marrow or lung transplantation, connective tissue diseases, inhalation of toxic substances, and drug toxicity (4). Three main BO entities are distinguished: postinfectious BO (PIBO); BO post lung transplantation; and BO after bone marrow transplantation (BMT) or hematopoietic stem cell transplantation (HSCT) (5). PIBO is the most common entity in children, and adenovirus (ADV) infection is the most common cause of PIBO (2, 3), especially ADV serotypes 3 and 7 (6, 7). ADV infection in children is endemic, epidemic or sporadic worldwide, most prevalent in winter and spring, and can be sporadic throughout the year. ADV plays a significant role in pediatric respiratory tract infections, especially in severe pneumonia, and it accounts for 3.5%–11% of childhood community-acquired pneumonias (6, 8–10).

BO is a clinical syndrome characterized by persistent wheezing and cough, and it is radiologically manifested by mosaic attenuation, bronchiolar dilation and wall thickening (11). BO may be classified in two main categories according to the pathology. The first one is proliferative BO, characterized by small airway lumen occlusion by granulation tissue polyps. The second category is constrictive BO, characterized by peri-bronchiolar fibrosis with various degrees of lumen constriction (12). In severe cases, there can be respiratory depression, decreased blood oxygen saturation, and even respiratory failure. Some children need frequent hospitalization and long-term home oxygen therapy, which seriously affects their quality of life and mental health. Some children even have a poor prognosis, and their lives could be in danger. However, the early identification of BO and the subsequent prompt intervention can obviously improve the prognosis of patients. Some studies have shown that fever, dyspnea, invasive mechanical ventilation, complications and persistent wheezing are independent risk factors for the development of BO in children after suffering from ADV pneumonia (13–15). In general, there is a relative scarcity of research on risk factors for BO, particularly after suffering from ADV pneumonia. Therefore, it is very valuable for us to find the independent risk factors for the development of BO after ADV pneumonia.

In this study, we collected and analyzed the clinical data of children with ADV pneumonia in our institution from March 2019 to March 2020 during the outbreak of ADV in 2019. The aim of this study was to determine the risk factors for the development of BO after suffering from ADV pneumonia.

## 2 Methods

### 2.1 Patients

**Figure 1:**
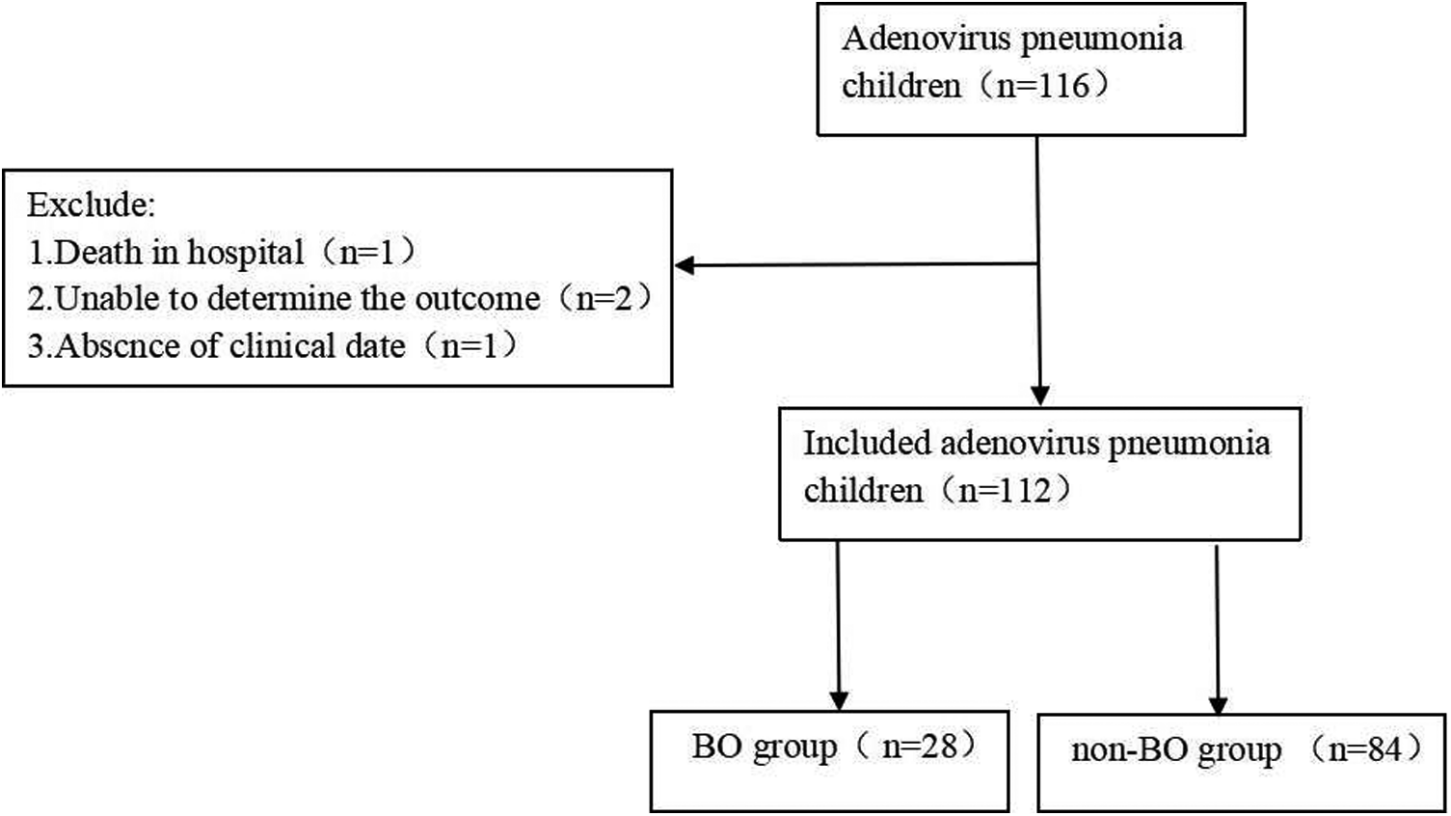
Patients included in this study. BO, bronchiolitis obliterans.

Our study is an observational cohort study that included 116 children suffering from ADV pneumonia. These children were hospitalized in our institution from March 2019 to March 2020. The ages of the children were between 1 month and 14 years old. Patients who had any underlying diseases, such as asthma, tuberculosis, congenital bronchopulmonary dysplasia, congenital heart disease, etc., were excluded. The children were followed up for at least 6 months to determine whether they developed BO. During the follow-up, patients who died, who lacked clinical data or in whom the outcome could not be determined were excluded (Figure 1). Finally, 112 children were included in this study. Among them, 28 children who did develop BO were defined as the “BO group”, and 84 children who did not develop BO were defined as the “non-BO group” (Figure 1).

Children were diagnosed with ADV pneumonia if they had signs of pneumonia on chest x-ray or CT and if they had a nasopharyngeal aspiration antigen test (D3 Ultra DFA Respiratory Virus Screening & ID Kit, Diagnostic Hybrids, Inc.) that was positive for ADV. The diagnostic criteria for PIBO caused by ADV pneumonia were as follows: (1) children had a history of pneumonia caused by ADV infection; (2) persistent or recurrent wheezing or cough, tachypnea, dyspnea, exercise intolerance, crackles for more than 6 weeks and poor response to bronchodilators (16); (3) high-resolution CT (HRCT) showed mosaic attenuation, dilated airways, and thickened bronchial walls; and (4) pulmonary function, showed small airway obstruction and limited or no response to bronchodilators. For four children aged 3 and above, we evaluated the forced expiratory volume in 1 s (FEV1) and maximum mid-expiratory flow at 75%–25% of forced vital capacity (MMEF75/25), FEV1 less than 80% and MMEF75/25 less than 65% suggested small airway obstruction, with limited or no response to bronchodilators. For the remaining 24 children under the age of 3, we assessed the peak expiratory flow-to-volume ratio (VPEF/VE) and time to peak expiratory flow-to-total expiratory time ratio (TPTEF/TE), and values less than 30% indicated the presence of small airway obstruction.

### 2.2 Data collection

Data from all patients were collected within one week of their disease onset. Follow-up data included months of age, sex, history of wheezing, atopy, ADV single or coinfection, tachypnea, wheezing, need for respiratory support, length of wheezing days, length of fever days, peak fever, hospitalization days, more than two lobes involved on CT, maximum CT value, C-reactive protein (CRP), white blood cell count (WBC), lymphocyte percentage (L), neutrophil percentage (N), hemoglobin (Hb), blood platelets (PLTs), alanine transaminase (ALT), aspartate aminotransferase (AST), lactic dehydrogenase (LDH), creatine kinase (CK), creatine kinase MB (CK-MB), creatinine (Cr), albumin (ALB), globulin (GLO), total bilirubin (TBIL), triglycerides (TG), total cholesterol (TC), procalcitonin (PCT), erythrocyte sedimentation rate (ESR), immunoglobulin G (IgG), immunoglobulin M (IgM), immunoglobulin A (IgA), complement 3 (C3), complement 4 (C4), T lymphocytes proportion, CD4+ T-cells proportion, CD8+ T-cells proportion, NK cells proportion, B lymphocytes proportion and CD4+/CD8+.

Tachypnea was defined as an increase in respiratory rate (more than 50 breaths/min in patients aged 2–12 months, more than 40 breaths/min in patients aged 1–5 years, more than 30 breaths/min in patients above 5 years old). Respiratory support was defined as the need for nasal catheter oxygen inhalation, continuous positive airway pressure (CPAP), or mechanical ventilation. The length of wheezing days was defined as the number of days the child had wheezing from the onset of ADV pneumonia to clinical remission before discharge.

Patients without evidence of bacterial, other viral or Mycoplasma pneumoniae (MP) infections were defined as single ADV infection. Patients who had positive bacterial growth on sputum culture were defined as coinfection with bacteria, and patients who tested positive for other viruses on respiratory viral nucleic acid testing were defined as coinfection with other viruses. Patients who have coinfection with MP should meet at least one of the following two criteria: (1) positive polymerase chain reaction (PCR) results for MP-DNA; (2) using the pneumonia Mycoplasma IgM and IgG detection kit to detect positive results for pneumonia Mycoplasma IgM and IgG by the chemiluminescence immunoassay method.

Whether more than two lobes were involved in CT and maximum CT values were determined by radiology experts in our institution (Philips Brilliance 64-slice spiral CT, Philips Medical Co., LTD.).

### 2.3 Statistical analysis

SPSS 24.0 software was used for statistical analysis. The measurement data were expressed by median (quartile distance) *M* (P25-P75) since the variance did not satisfy a normal distribution by the Kolmogorov‒Smirnov (K-S) test, and the comparison between groups was performed by the Mann‒Whitney *U* test. The enumeration data were represented by the number of cases (percentage) *n* (%), and the comparison between groups was performed by the *χ^2^* test. All of the risk factors were analyzed by a univariate logistic regression test, and *P* < 0.05 indicated that the difference was statistically significant. Variables that had a *P* value < 0.05 in the univariate logistic analysis were included in the multivariate logistic regression analysis using stepwise forward selection to determine the independent risk factors for BO. The inclusion criterion for factors was *P* < 0.05, and the sensitivity and specificity of the predictive model constructed using logistic regression analysis were evaluated using the receiver operating characteristic (ROC) curve analysis.

## 3 Results

### 3.1 Comparison of the general data between the two groups

A total of 116 patients diagnosed with ADV pneumonia were enrolled, and 112 children (77 boys and 35 girls) were included in our study. After a 6-month follow-up, 28 children (25%) did develop BO, which we defined as the “BO group”, and 84 children (75%) did not develop BO, which we defined as the “non-BO group” (Figure 1). The general characteristics of the two groups, including months of age, sex, history of wheezing and atopy, were compared, and no significance was found (Table 1).

**Table 1:** Comparisons of general characteristics between the two groups.

	BO group (*n* = 28)	Non-BO group (*n* = 84)	Statistics (*Z*/χ^2^)	*P* value
Months of age	16 (11.25–30.75)	25 (12–50.5)	−1.768	0.077
Sex (male/female)	21/7	56/28	0.679	0.410
History of wheezing (%)	5 (17.9)	6 (7.1)	2.722	0.099
Atopy (%)	4 (14.3)	12 (14.3)	0.000	1.000

Values are presented as median (quartile distance) *M* (P25–P75) or the number of cases (percentage) *n* (%). BO, bronchiolitis obliterans.

### 3.2 Comparison of the clinical data between the two groups

Clinical characteristics of the two groups were compared (Table 2). Among all the clinical characteristics, coinfection with other viruses, coinfection with MP, tachypnea, wheezing, respiratory support, length of wheezing days, length of fever days and hospitalization days were found to be significantly different between the two groups (*P *< 0.05).

**Table 2:** Univariate comparisons of the clinical characteristics.

	BO group (*n* = 28)	Non-BO group (*n* = 84)	Statistics (*Z*/*χ*^2^)	*P* value
Single adenovirus infection (%)	7 (25.0)	17 (20.2)	0.283	0.595
Coinfection with bacteria (%)	9 (32.1)	32 (38.1)	0.321	0.571
Coinfection with other viruses (%)	9 (32.1)	11 (13.1)	5.194	0.023*
Coinfection with MP (%)	10 (35.7)	48 (57.1)	3.862	0.049*
Tachypnea (%)	15 (53.6)	15 (17.9)	13.659	<0.001*
Wheezing (%)	23 (82.1)	33 (39.3)	15.429	<0.001*
Respiratory support (%)	22 (78.6)	23 (27.4)	22.895	<0.001*
Length of wheezing days(d)	9.50 (4.25–17.75)	0.00 (0.00–5.00)	−4.778	<0.001*
Length of fever days(d)	10.00 (7.00–13.75)	8.00 (6.00–12.00)	−1.992	0.046*
Peak temperature (°C)	40.00 (39.50–40.38)	40.00 (39.48–40.20)	−0.606	0.545
Hospitalization days (d)	12.50 (10.00–17.00)	9.00 (7.00–11.75)	−3.616	<0.001*
≥2 lobes involved on CT (%)	26 (92.9)	40 (76.9)	3.201	0.074
Maximum CT value	30.00 (24.25–34.75)	32.50 (23.00–40.75)	−0.636	0.521

Values are presented as median (quartile distance) *M* (P25–P75) or the number of cases (percentage) *n* (%). BO, bronchiolitis obliterans.

* Compared with non-BO group, *P* < 0.05.

The laboratory examination results of the two groups were also compared (Table 3). Among all the laboratory examination results, AST, LDH, CK-MB, PCT and B lymphocyte proportions in the BO group were found to be significantly higher than those in the non-BO group (*P *< 0.05). IgG, IgM, C3, T lymphocyte proportion, CD4+ T-cell proportion and CD8+ T-cell proportion were found to be significantly lower than those in the non-BO group (*P *< 0.05).

**Table 3:** Univariate comparisons of the laboratory examination characteristics.

	BO group (*n* = 28)	Non-BO group (*n* = 84)	Statistics (*Z*)	*P* value
CRP (mg/L)	8.00 (8.00–16.00)	8.00 (8.00–25.75)	−0.385	0.700
WBC (×10^9^/L)	9.00 (7.55–12.18)	9.51 (7.15–13.23)	−0.168	0.867
L (%)	27.90 (18.30–41.73)	34.85 (25.38–42.90)	−1.640	0.101
N (%)	67.55 (45.50–77.30)	53.85 (46.58–69.15)	−1.878	0.060
Hb (g/L)	115.50 (106.50–123.00)	117.50 (110.00–123.00)	−0.662	0.508
PLTs (×10^9^/L)	261.50 (180.25–369.25)	250.00 (181.50–363.25)	−0.057	0.954
ALT (U/L)	25.50 (17.25–43.00)	20.00 (14.00–31.75)	−1.583	0.113
AST (U/L)	72.50 (40.25–97.25)	40.00 (30.25–72.25)	−2.265	0.024*
LDH (U/L)	763.00 (587.00–1,257.25)	477.00 (327.50–745.50)	−3.343	0.001*
CK (U/L)	136.50 (68.75–437.75)	74.00 (52.25–157.50)	−1.955	0.051
CKMB (U/L)	36.00 (28.25–52.50)	28.00 (23.00–36.00)	−2.768	0.006*
Cr (umol/L)	28.00 (25.00–36.00)	33.00 (26.00–40.00)	−1.931	0.054
ALB (g/L)	36.35 (34.68–41.15)	39.50 (35.40–42.58)	−1.112	0.266
GLO (g/L)	26.05 (24.25–30.98)	26.25 (23.90–30.85)	−0.316	0.752
TBIL (umol/L)	3.85 (2.50–4.83)	3.65 (2.83–5.28)	−0.434	0.665
TG (mmol/L)	1.75 (1.22–2.17)	1.51 (1.05–2.14)	−0.971	0.332
TC (mmol/L)	3.43 (2.54–4.19)	3.39 (2.92–3.88)	−0.386	0.699
PCT (ng/ml)	0.66 (0.17–1.48)	0.17 (0.07–0.54)	−3.062	0.002*
ESR (mm/h)	27.00 (23.00–37.50)	33.00 (17.00–54.50)	−0.595	0.552
IgG (g/L)	7.24 (5.48–10.46)	8.77 (6.67–12.00)	−2.055	0.040*
IgM (g/L)	0.92 (0.69–1.31)	1.32 (0.88–2.09)	−2.510	0.012*
IgA (g/L)	0.49 (0.32–0.71)	0.58 (0.40–1.14)	−1.958	0.050
C3 (g/L)	0.90 (0.73–1.20)	1.08 (0.90–1.29)	−2.350	0.019*
C4 (g/L)	0.26 (0.18–0.35)	0.25 (0.18–0.33)	−0.501	0.616
T lymphocyte (%)	50.22 (41.10–58.25)	61.93 (52.89–71.29)	−4.015	<0.001*
CD4+ *T*-cells (%)	26.84 (19.21–33.93)	33.86 (26.40–41.23)	−2.910	0.004*
CD8+ *T*-cells (%)	20.56 (14.98–25.70)	24.93 (20.90–30.78)	−2.957	0.003*
NK cell (%)	10.07 (6.25–14.50)	9.30 (6.10–13.49)	−0.202	0.840
B lymphocyte (%)	33.39 (21.07–43.79)	20.28 (13.10–31.34)	−3.995	<0.001*
CD4+/CD8+	1.32 (0.90–1.76)	1.36 (0.97–1.81)	−0.185	0.853

Values are presented as median (quartile distance) *M* (P25–P75). BO, bronchiolitis obliterans; CRP, C-reactive protein; WBC, white blood cell count; L, lymphocytes percentage; N, neutrophil percentage; Hb, hemoglobin; PLT, blood platelet; ALT, alanine transaminase; AST, aspartate aminotransferase; LDH, lactic dehydrogenase; CK, creatine kinase; CK-MB, creatine kinase MB; Cr, creatinine; ALB, albumin; GLO, globulin; TBIL, total bilirubin; TG, triglyceride; TC, total cholesterol; PCT, procalcitonin; ESR, erythrocyte sedimentation rate; IgG, immunoglobulin G; IgM, immunoglobulin M; IgA, immunoglobulin A; C3, complement 3; C4, complement 4.

* Compared with non-BO group, *P* < 0.05.

### 3.3 Univariate logistic regression analysis of the risk factors for the development of BO

Nineteen factors of clinical characteristics and laboratory examination results were found to be significantly different between the BO group and the non-BO group, and they were analyzed by univariate logistic regression analysis. Finally, 14 risk factors were found to have statistical significance for the development of BO after ADV pneumonia (*P *< 0.05): coinfection with other viruses, tachypnea, wheezing, respiratory support, length of wheezing days, length of fever days, hospitalization days, LDH levels, PCT values, C3 values, CD4+ T-cell proportion, CD8+ T-cell proportion, T lymphocyte proportion and B lymphocyte proportion (Table 4).

**Table 4:** Univariate logistic regression analysis of risk factors for the development of BO.

	*β*	SE	Wald *χ*^2^	*P*	OR	95% CI
Coinfection with other viruses	1.145	0.518	4.888	0.027*	3.144	1.139–8.677
Coinfection with MP (%)	−0.875	0.452	3.754	0.053	0.417	0.172–1.010
Tachypnea	1.669	0.474	12.396	<0.001*	5.308	2.096–13.441
Wheezing	1.961	0.542	13.112	<0.001*	7.109	2.459–20.553
Respiratory support	2.275	0.522	19.023	<0.001*	9.725	3.499–27.027
Length of wheezing days	0.107	0.043	6.216	0.013*	1.113	1.023–1.212
Length of fever days	0.107	0.043	6.216	0.013*	1.113	1.023–1.212
Hospitalization days	0.124	0.045	7.543	0.006*	1.132	1.036–1.236
AST	0.000	0.002	0.010	0.922	1.000	0.996–1.005
LDH	0.001	0.000	8.564	0.003*	1.001	1.000–1.002
CKMB	0.013	0.009	2.111	0.146	1.013	0.995–1.031
PCT (*n* = 109)	0.557	0.259	4.644	0.031*	1.746	1.052–2.899
IgG	−0.087	0.053	2.657	0.103	0.917	0.825–1.018
IgM	−0.646	0.332	3.777	0.052	0.524	0.273–1.006
C3	−1.971	0.791	6.217	0.013*	0.139	0.030–0.656
T lymphocyte	−0.078	0.020	14.808	<0.001*	0.925	0.889–0.962
CD4+ T-cells	−0.071	0.026	7.611	0.006*	0.931	0.885–0.980
CD8+ T-cells	−0.097	0.034	7.930	0.005*	0.908	0.849–0.97
B lymphocyte	0.072	0.019	13.749	<0.001*	1.074	1.034–1.116

AST, aspartate aminotransferase; LDH, lactic dehydrogenase; CK-MB, creatine kinase MB; PCT, procalcitonin; IgG, immunoglobulin G; IgM, immunoglobulin M; C3, complement 3; SE, standard error; OR, odds ratio; CI, confidence interval.

* Compared with non-BO group, *P* < 0.05.

### 3.4 Multivariate logistic regression analysis of risk factors for the development of BO

The 14 risk factors found with statistical significance in the univariate logistic regression analysis were analyzed by multivariate logistic regression analysis, and the independent risk factors for BO were indicated as respiratory support (OR 6.772, 95% CI 2.060–22.260, *P *= 0.002), length of wheezing days (OR 1.112, 95% CI 1.040–1.189, *P *= 0.002) and LDH levels (OR 1.002, 95% CI 1.000–1.003, *P *= 0.012) (Table 5).

**Table 5:** Multivariate logistic regression of independent risk factors for the development of BO.

	*β*	SE	Wald *χ*^2^	*P*	OR	95% CI
Respiratory support	1.913	0.607	9.927	0.002	6.772	2.060–22.260
Length of wheezing days	0.106	0.034	9.675	0.002	1.112	1.040–1.189
LDH	0.002	0.001	6.329	0.012	1.002	1.000–1.003
Constant	−3.982	0.853	21.785	0.000	0.019	–

LDH, lactic dehydrogenase; SE, standard error; OR, odds ratio; CI, confidence interval.

### 3.5 The value of the predictors

**Figure 2:**
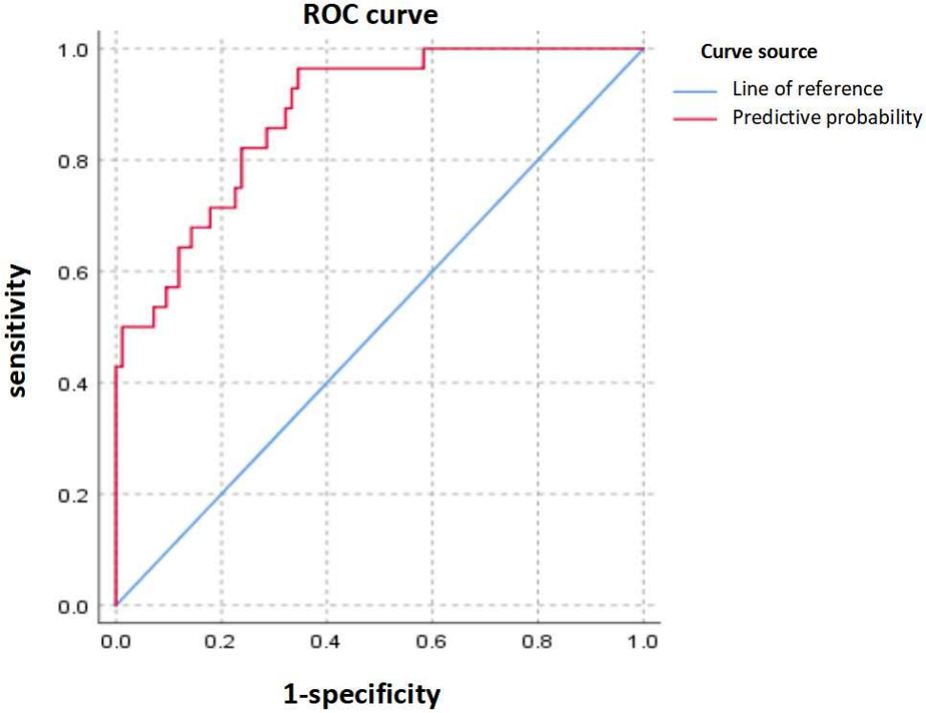
Receiver operating characteristic (ROC) curve. The upper curve represents ROC curve for the prediction probability model for independent risk factors for BO, and the area under ROC curve = 0.870 (95% CI 0.801–0.939).

The predictive model constructed using logistic regression analysis was evaluated by the ROC curve, which demonstrated excellent diagnostic accuracy with an area under the curve (AUC) of 0.870 (95% CI, 0.801–0.939; *P* < 0.001) (Figure 2). Additionally, the ROC curve analysis revealed a standard error of 0.035, a maximum Youden's index of 0.608, a sensitivity of 0.929, and a specificity of 0.679.

## 4 Discussion

Most cases of BO in children is postinfectious, especially after an ADV infection (2). In this study, we found that a quarter of children developed BO after suffering from ADV pneumonia. Zhong et al. found that 24.5% of severe ADV pneumonia patients developed BO (13), which is similar to our results.

In our study, three independent risk factors for the development of BO after ADV pneumonia were detected. Among them, two risk factors were found for the first time, with the first being the length of wheezing days. Up until now, the correlation between the length of wheezing days and the development of BO after ADV pneumonia has not been analysed in the literature. However, Yu et al. found that persistent wheezing was one of the strongest independent risk factors for PIBO after ADV pneumonia (14). We speculate that the extended length of wheezing days may be a clinical expression of the irreversibility of airway obstruction. Raised LDH levels are a second independent risk factor for the development of BO after suffering from ADV pneumonia, which we found for the first time. Some studies have found that the LDH level is an independent risk factor for the development of BO after MP pneumonia (17–19), but there are no reports in ADV-PIBO. Although the study of Zou et al. also suggests that pretreatment LDH was significantly higher in children with severe ADV pneumonia who developed PIBO than in those who did not, it was not an independent risk factor (20). The increase in LDH levels suggested severe lung injury, which may induce excessive abnormal repair, may play a role in the pathogenesis of BO. In addition to the above two independent factors, our study also showed that respiratory support is a third independent risk factor for the development of BO after suffering from ADV pneumonia. A number of studies have also shown that mechanical ventilation is an independent risk factor for the development of BO after suffering from ADV pneumonia (21–23), consistent with our results. We speculate that, on one hand, the need for mechanical ventilation suggests a more severe airway obstruction in the patients. On the other hand, mechanical ventilation-induced lung injury may induce excessive abnormal repair, potentially playing a role in the development of BO.

Furthermore, the T lymphocyte proportion, CD4+ T-cell proportion and CD8+ T-cell proportion in the BO group were found to be significantly lower than those in the non-BO group in our study, and they were found to have statistical significance for the development of BO by univariate logistic regression analysis (*P *< 0.05). It is well known that CD8+ T cells exert potent cytotoxic effects against viruses, and CD4+ T cells can assist B lymphocytes in differentiating into plasma cells to produce antibodies to neutralize viruses. We speculate that the reduction in CD4+ T cells and CD8+ T cells leads to a decrease in the ability to clear ADV, and a persistent ADV infection may lead to sustained and irreversible damage to the small airways. In addition, the proportion of coinfection with other viruses in the BO group was found to be significantly higher than that in the non-BO group in our study, and it was found to be statistically significant by univariate logistic regression analysis (*P *< 0.05). We speculate that this is because in addition to ADV, other viral infections, such as measles virus, respiratory syncytial virus (RSV), herpes simplex virus, influenza virus, parainfluenza type 3, human immunodeficiency virus type 1 and cytomegalovirus, can also induce BO (4, 13, 19). Therefore, coinfection with other viruses may exacerbate the progression of BO. However, because there is no literature report of coinfection with other viruses being a risk factor for BO, a larger sample size is still needed for further confirmation.

Our study has several limitations. First, the number of children with BO was low in our study, which is attributed to the rarity of BO. Second, this study was a single-center study, and the results may vary across different institutions. Finally, our study did not identify the type of ADV, and the type of ADV which specifically may be related to the development of BO.

In conclusion, a quarter of children developed BO after suffering from ADV pneumonia in our study, and moreover, children who have needed respiratory support, had a longer length of wheezing days or had higher LDH levels are more likely to develop BO.

## Data Availability

The original contributions presented in the study are included in the article/Supplementary Material, further inquiries can be directed to the corresponding author.
